# Time-course effects of functional fitness sessions performed at different intensities on the metabolic, hormonal, and BDNF responses in trained men

**DOI:** 10.1186/s13102-022-00412-6

**Published:** 2022-02-08

**Authors:** Ramires Alsamir Tibana, Ivo Vieira de Sousa Neto, Nuno Manuel Frade de Sousa, Wellington Martins dos Santos, Jonato Prestes, João Henrique Falk Neto, Fábio H. Dominski, Michael D. Kennedy, Fabricio Azevedo Voltarelli

**Affiliations:** 1 Graduate Program in Health Sciences, Faculty of Medicine, Federal University of Mato Grosso (UFMT), Cuiabá, Brazil; 2 Laboratory of Molecular Analysis, Graduate Program of Sciences and Technology of Health, University of Brasilia, Brasilia, Brazil; 3 Laboratory of Exercise Physiology, Faculty Estacio of Vitoria, Vitoria, Brazil; 4 Graduate Program On Physical Education, Catholic University of Brasilia, Brasilia, Brazil; 5 Athlete Health Lab, University of Alberta, Edmonton, AB Canada; 6 Laboratory of Sport and Exercise Psychology, Human Movement Sciences Graduate Program, College of Health and Sport Science of the Santa Catarina State University (UDESC), Florianópolis, Brazil

**Keywords:** CrossFit, High-intensity functional training, Rating perceived exertion, Endocrine system, Metabolism, Neurotrophin

## Abstract

**Background:**

To investigate the time-course effects of a self-regulated training session (performed at an rating perceived exertion of 6/10), all-out session, and a control session on the metabolic, hormonal, and brain derived neurotrophic factor (BDNF) responses in Functional-Fitness (FFT) participants.

**Methods:**

In a randomized, crossover fashion, eight healthy males (age 28.1 ± 5.4 years old; body mass 77.2 ± 4.4 kg; VO_2max_: 52.6 ± 4.6 mL.(kg.min)^−1^; 2000 m rowing test 7.35 ± 0.18 min; 1RM back squat 135.6 ± 21.9 kg) performed a FFT session under two different conditions: all-out, or with the intensity controlled to elicit an rating perceived exertion (RPE) of 6 in the Borg 10-point scale (RPE6). A control session (no exercise) was also completed. Metabolic (lactate and creatine kinase), hormonal (testosterone and cortisol), and BDNF responses were assessed pre, post-0 h, 1 h, 2 h and 24 h after the sessions.

**Results:**

Creatine kinase concentrations were significantly higher (*p* ≤ 0.05) after 24 h for both training sessions. Total and free testosterone concentrations were lower post-2 h for all-out when compared to the RPE6 session (*p* ≤ 0.05). Serum cortisol concentration increased post-0 h (*p* = 0.011) for RPE6 and post-0 h (*p* = 0.003) and post-1 h (*p* = 0.030) for all-out session when comparing to baseline concentrations. BDNF was significantly higher (*p* = 0.002) post-0 h only for the all-out session when compared to baseline. A positive correlation between blood lactate concentrations and BDNF (r = 0.51; *p* = 0.01) was found for both effort interventions.

**Conclusions:**

A single FFT session when performed in all-out format acutely increases the concentrations of serum BDNF. However, physiological stress markers show that the all-out session requires a longer recovery period when compared to the RPE6 protocol. These findings can be helpful to coaches and practitioners design FFT session.

## Introduction

Functional fitness training (FFT) (a.k.a. CrossFit) involves the performance of exercises that comprise whole body, and that are executed in multiple planes of motion [1]. The FFT sessions can be designed to challenge various physiological systems at the same time [2], through the use of gymnastics, weightlifting, and cardiovascular exercises [3], and thus, have been shown to improve multiple fitness components concomitantly. Practitioners commonly perform 3–5 whole body training sessions per week, and while the selection of exercises depends on which fitness components are being targeted [1], most sessions dedicate a period of time to metabolic conditioning [4].

One of the potential reasons why the physiological responses to metabolic conditioning of FFT are not yet well understood lies in the fact that the sessions can vary significantly. The protocols might differ in their duration (2–30 min), exercise modality and selection (cardiovascular, gymnastic, and/or weightlifting exercises), method [for time or as many rounds as possible (AMRAP)], and intensity (absolute or relative load) [5]. Many of these sessions are performed as all-out efforts, where the goal is to complete the task in the shortest amount of time possible or to complete the highest amount of work in a set period of time [2, 6, 7]. As many of these sessions are performed at a high intensity, previous research has shown that the metabolic conditioning sessions of FFT resulted in increased acute oxidative stress [8]; high metabolic, inflammatory [3], and cardiovascular responses; elevated perceived exertion [9]; and increased sympathetic nervous system markers (i.e., epinephrine and norepinephrine) [10]. As a result of the increases in oxidative and inflammatory markers, and the extreme effort associated with FFT, some studies have raised concerns about a tendency for the development of symptoms of overtraining in functional fitness practitioners [8]. To address this issue, previous studies have demonstrated the effectiveness of utilizing the rating of perceived exertion (RPE) to control the intensity of these sessions, and consequently, the physiological responses to exercise during [4] and after [2] a FFT session.

The distinct modes, intensity, and duration of exercises that can be manipulated in FFT sessions, along with differences in individual responsiveness to the sessions, may lead to different hormonal responses post-FFT sessions [11]. Such changes in hormonal profiles following a session might have important implications. Previous studies have revealed that long periods of hormonal disturbances are likely to lead to impairments in performance, inflammatory conditions, and increased muscle fatigability [12–14]. Similarly, while transient increases in creatine kinase, and cortisol immediately following a training session are expected, chronically high levels are not desirable [4]. In this context, changes in hormonal responses can provide important information in detecting early signs of non-functional overreaching. Understanding the time-course endocrine response to FFT sessions performed at different intensities, therefore, might assist in ensuring optimal training prescription.

In addition to its effects on hormonal concentrations, FFT has been demonstrated to elicit important alterations in other biomarkers. The brain derived neurotrophic factor (BDNF) is a key biomarker that stimulates neurogenesis, neuron survival, and modulates the differentiation of cells developed in the hippocampus, which can be essential for cognition, memory [15] and consequently neuroplasticity improvement [16]. Murawska-Cialowicz et al. [17] revealed that 3 months of FFT training resulted in a significant increase in resting BDNF levels in male and female participants. Indeed, a single FFT session is a potent stimulus that leads to an acute increase in serum BDNF concentrations [18]. Of interest, prior study has demonstrated that blood lactate produced during exercise is correlated with BDNF production [19] and recently a systematic review with meta-analysis considered that FFT session normally causes a substantial metabolic stress, leading to metabolite accumulation (e.g., lactate up to 18 mmol/L) [20]. Therefore, understanding the time-course of changes in peripheral BDNF levels and the possible relationship with lactate following FFT sessions performed at different intensities is an important pursuit.

Considering that the adaptations elicited during a training program result from the summation of training bouts, understanding the role that the intensity of FFT protocols have on neurotrophin, metabolic and hormonal biomarkers in a time-dependent manner is an important step to understand these adaptive mechanisms. Specifically, this might assist in clarifying the key mediators by which FFT exerts beneficial or maladaptive effects. Assessing the response of different biomarkers to the training stimulus might help in understanding the overall effectiveness of FFT sessions performed at distinct intensities, and therefore, assist in optimizing training programs. Thus, the purpose of the present study was to investigate the acute time-course of metabolic, hormonal and BDNF responses following two FFT sessions performed at different intensities (RPE6 and all-out). It is hypothesized that a higher intensity will lead to a more pronounced hormonal and metabolic response, with the lower intensity session (RPE6) showing a reduced magnitude of endocrine response to an FFT session.

## Methods

### Subjects

Eight male subjects (age 28.1 ± 5.4 years old; body mass 77.2 ± 4.4 kg; VO_2max_: 52.6 ± 4.6 mL.(kg.min)^−1^; 2000 m rowing test: 7.35 ± 0.18 min; 1RM back squat: 135.6 ± 21.9 kg) were recruited. All subjects were free of injury or known illnesses, were not using performance enhancing drugs, and had more than 12 months of FFT experience (3.8 ± 1.4 years, 1.5–6 years of experience). Participants were advised to sleep six to eight hours the night before the tests, maintain regular nutritional and hydration habits, avoid intense exercise 48 h prior to the sessions, as well as avoid smoking, alcohol, and caffeine consumption 24 h before a session. All subjects provided informed consent, and the study was approved by the University Research Ethics Committee for Human Use (2.698.225/Universidade Estácio de Sá/UNESA/RJ and ethics ID Pro00110581) and conformed to the Helsinki Declaration on the use of human participants for research.

### Experimental design

**Fig. 1:**
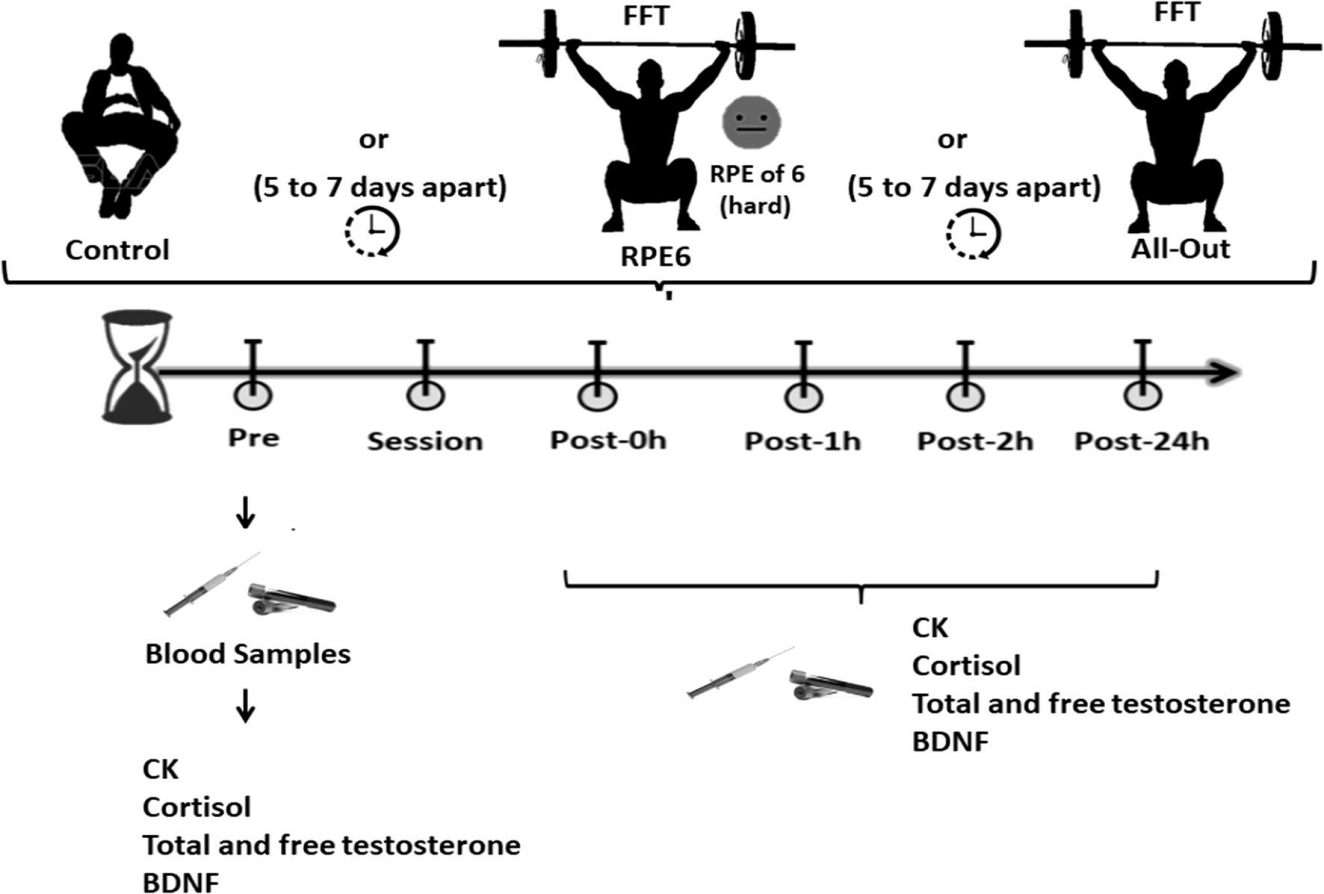
Schematic study design and timeline used to examine the time-course effects of RPE6, all-out and control sessions on metabolic (lactate and creatine kinase), hormonal (cortisol, total and free testosterone) and brain derived neurotrophic factor (BDNF) response in practitioners of FFT

In this study, the participants performed a FFT session under two different conditions, either with the intensity controlled based on an RPE of 6 (RPE6) or as an all-out effort (all-out), in addition to a control session (CON). The metabolic and hormonal responses to the different conditions were the dependent variables. These responses were assessed prior to the start of the session, immediately post, and 1 h, 2 h and 24 h after the sessions, to compare the acute effects of a metabolic conditioning FFT session performed with different intensities (Fig. 1).

**Fig. 2:**
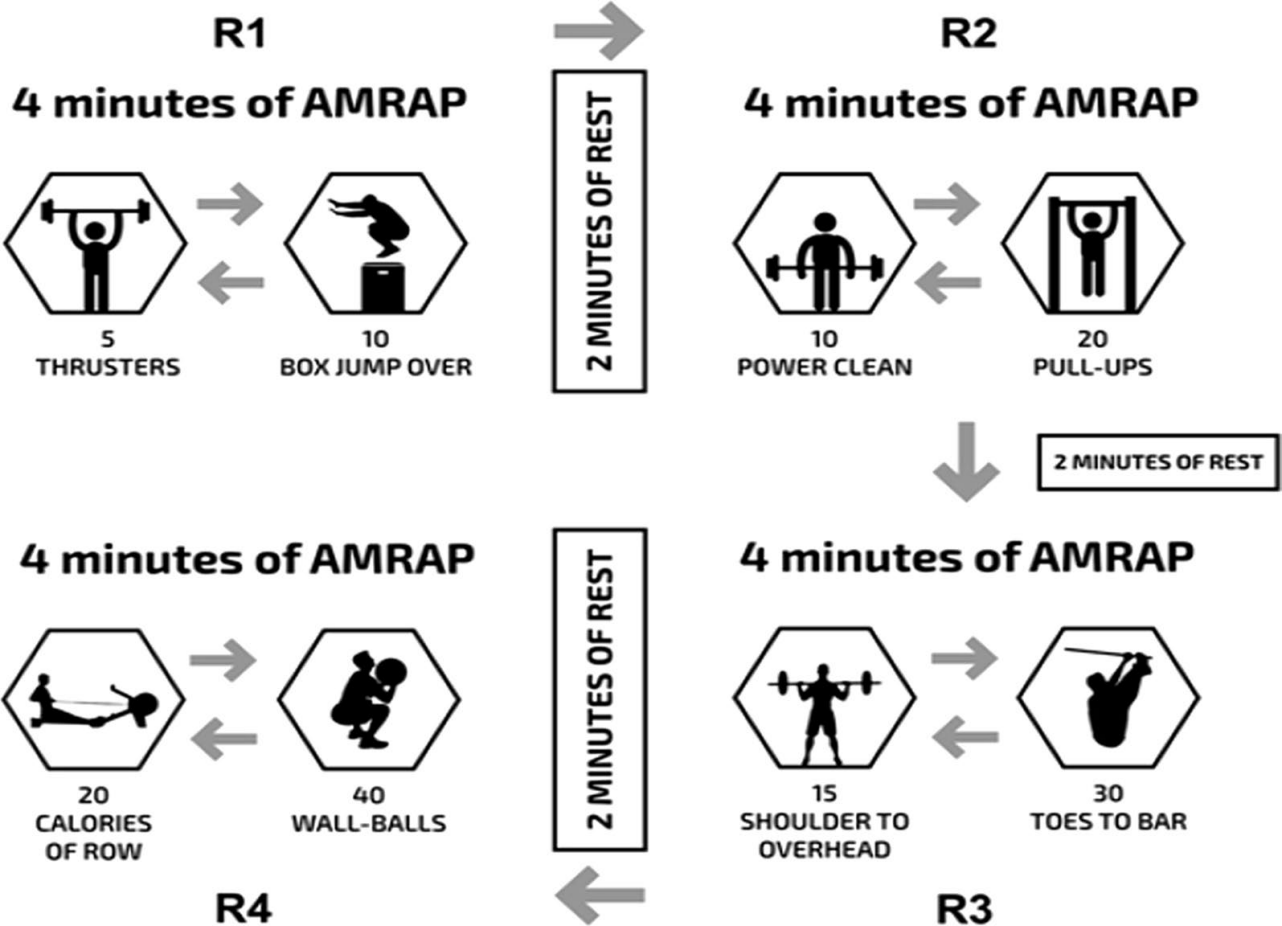
Description of the metabolic conditioning sessions (Tibana Test). AMRAP, as many rounds as possible

The subjects completed a metabolic conditioning training session (five to seven days apart) in a randomized fashion under two different conditions: (a) all-out or (b) intensity-controlled, based on an RPE6 (hard) on a modified version of the Borg CR-10 scale (RPE6) [2]. A control session, where the participants were instructed to spend 22 min in a sitting position, without any type of exercise, was also performed. The metabolic conditioning training session was the Tibana Test [2], which involved the completion of four different bouts of work, each separated by 2 min of rest (Fig. 2). The rounds consisted of 4 min of as many rounds as possible (AMRAP) of five thrusters (60 kg) and 10 box jumps over (round 1); 4 min of AMRAP of 10 power clean (60 kg) and 20 pull-ups (round 2); 4 min of AMRAP of 15 shoulder to overhead (60 kg) and 30 toes to bar (round 3); and 4 min of AMRAP of 20 calories of rowing and 40 wall ball (9 kg; round 4).

During the all-out condition, the subjects were instructed to complete the maximum number of repetitions possible for each round. In the RPE6 condition, they performed the same conditioning session, but were told to self-regulate the intensity of their effort based on a perception of effort of 6 out of 10, on an adapted version of the Borg CR-10 scale [21, 22]. To achieve this, subjects were instructed to take more breaks if needed, or to pace themselves in the execution of their exercises to keep the perception of effort at the desired level. No changes to the weights were performed during the sessions. The adapted Borg CR-10 scale was printed and available to the participants as a visual reminder of the prescribed target intensity. This strategy has been shown to be successful in ensuring the athletes perform their FFT sessions within the desired intensity, based on previously published work [2, 7]. During each metabolic condition training session and the control session, blood samples were collected from the antecubital vein to analyze changes in the concentrations of creatine kinase, cortisol, total testosterone, free testosterone and BDNF.

### Blood samples collected, hormonal and BDNF analysis

Blood samples were collected immediately before (pre) and immediately (post-0 h), 1 h (post-1 h), 2 h (post-2 h) and 24 h (post-24 h) after the FFT and control sessions by venipuncture from the antecubital vein. The samples were collected in 5-mL evacuated tubes (Vacutainer; Becton, Dickinson and Company, Franklin Lakes, NJ, USA). The tubes were refrigerated for 1 h and samples were centrifuged at 2.900 RPM for 15 min at 4 °C, and the resultant serum divided into several aliquots, and frozen at − 80 °C until analysis. Hormonal analyses were performed using a commercial PCR kit lot 167404 (Roche), specific for humans in an automated device (Cobas E601—Roche) for PCR by the electrochemiluminescence method. Serum was analyzed for BDNF using a commercially available enzyme-linked immunosorbent assay (ELISA) kit according to manufacturer’s instructions (MyBioSource Inc., San Diego, CA, USA). All samples were determined in duplicate to guarantee the precision of the results. Detection range of this method was 31.25–2000 pg/mL, sensitivity of this methods was 18.75 pg/mL, with an intraassay coefficient of 4% and an inter-assay coefficient of 8%. All analyses were performed in the Immune Gerontology/ Molecular Biology Laboratory of Applied Exercise at the University.

### Creatine kinase analysis

Whole-blood creatine kinase activity was assessed from a single fingertip capillary sample with the subject in a seated position. After pre-warming the hand, a sample of blood (30 μL) was obtained and analyzed using a colorimetric assay procedure (Reflotron, Boehringer Mannheim, Germany). Before each testing session, quality control (calibration) measurements were undertaken according to the manufacturer’s recommendations. The ‘‘normal’’ reference range for creatine kinase activity, as provided by the manufacturer, is 24–195 U/L.

### Blood lactate

Standard procedures were followed for blood lactate collection, management, and analysis according to Falk Neto et al. [2]. Capillary blood samples were collected through a transcutaneous puncture on the medial side of the tip of the middle finger using a disposable hypodermic lancet. Blood lactate concentration was determined by photometric reflectance on a validated Portable Accutrend Plus system (Roche, Sao Paulo, Brazil).

### Statistical analysis

The data are presented as means and 95% confidence intervals (CI). Shapiro–Wilk tests were applied to assess the normal distribution of the variables assessed. In case of non-normal distribution, the variables were log transformed before analysis. Repeated measures ANOVA was used to compare creatine kinase, and hormonal status between the FFT sessions and the control session. Tukey’s post-hoc test was applied in the event of a significant main effect. Repeated measures ANOVA was also used to compare creatine kinase and hormonal concentrations between pre- values and post-FFT sessions in different time points. Lastly, percentage of change from baseline of creatine kinase, and hormonal concentrations was calculated for the different time points and repeated measures ANOVA was also used to compare creatine kinase, and hormonal changes between functional fitness sessions and control session. Cohen’s d effect sizes (ES) were calculated using the Cohen´s convention [23] to evaluate the magnitude of the change of creatine kinase and hormonal concentrations during the functional fitness and control sessions (ES ≤ 0.20 represents a small ES; 0.50 a moderate ES; 0.80 a large effect size). Simple Pearson’s r correlations were used to determine the associations between the hormonal responses and blood lactate concentration and RPE after the FFT sessions and the control condition. The magnitude of the correlations was classified as: r ≤ 0.1 trivial; 0.1 < r ≤ 0.3 small; 0.3 < r ≤ 0.5 moderate; 0.5 < r ≤ 0.7 large; 0.7 < r ≤ 0.9 very large; r > 0.9 almost perfect [24]. The level of significance was *p* ≤ 0.05 and SPSS version 20.0 (Somers, NY, USA) software was used.

## Results

Participants completed a greater number of repetitions (214.4 ± 18.6 repetitions) during the all-out session when compared to the RPE6 session (190.5 ± 12.5 repetitions). Blood lactate concentration and RPE were also higher after the all-out session (18.9 ± 3.9 mmol/L; RPE: 9.6 ± 0.7) than the RPE6 session (12.8 ± 3.2 mmol/L; RPE: 6.2 ± 0.8). An in-depth discussion of these results and its implications has already been published [4].

Table 1 presents the hormonal responses pre- and post-functional fitness sessions. Considering the baseline values, only the cortisol concentration pre- all-out session was greater (*p* = 0.048) than the control session. Creatine kinase concentration was greater 0 h (*p* = 0.038), 1 h (*p* = 0.010), 2 h (*p* = 0.011) and 24 h (*p* = 0.041) after the RPE6 and 0 h (*p* = 0.001), 1 h (*p* = 0.002), 2 h (*p* = 0.015) and 24 h (*p* = 0.058) after the all-out sessions. When compared to baseline, serum cortisol concentration was greater post-0 h (*p* = 0.011) for RPE6 and post-0 h (*p* = 0.003) and post-1 h (*p* = 0.030) for all-out session. For the all-out session, cortisol concentration post-24 h was significantly less (*p* = 0.010) than pre- values. Total testosterone and free testosterone were significantly greater (*p* ≤ 0.05) post-0 h for RPE6 (p = 0.007 for total testosterone and *p* = 0.010 for free testosterone) and all-out (*p* = 0.005 for total testosterone and *p* = 0.003 for free testosterone) sessions when comparing to baseline concentrations. However, total testosterone and free testosterone were significantly lower (*p* = 0.009 for total testosterone and *p* = 0.010 for free testosterone) post-1 h comparing to baseline concentrations only for RPE6 session. BDNF concentration was higher (*p* = 0.002) post-0 h only for the all-out session when comparing to baseline concentrations. However, BDNF was significantly lower post-24 h after the all-out (*p* = 0.042) and RPE6 (*p* = 0.032) sessions, when compared to baseline values.

**Table 1:** Creatine kinase and hormonal responses pre- and post-functional fitness sessions with self-regulation of intensity (RPE6) and ALL-OUT and control session [mean (95% CI)]

	Pre	Post-0 h	Post-1 h	Post-2 h	Post-24 h
Control					
Creatine kinase, U/L	282 (159–404)	281 (161–402)	287 (149–424)	279 (151–407)	270 (134–405)
Cortisol, ug/dL	8.9 (7.0–10.7)	8.7 (7.0–10.5)	7.6 (6.4–8.9)	8.3 (7.2–9.4)	7.1 (6.1–8.1)
Total testosterone, ng/dL	571 (496–646)	583 (503–663)	534 (456–611)	565 (497–633)	526 (455–597)
Free testosterone, ng/dL	20.3 (17.6–23.0)	20.9 (18.3–23.5)	18.7 (16.1–21.4)	19.6 (17.5–21.7)	18.3 (16.2–20.3)
BDNF, pg/mL	374 (167–581)	291 (67–650)	344 (31–669)	508 (122–898)	239 (167–311)
RPE6					
Creatine kinase, U/L	318 (100–536)	410 (111–710)^†^	359 (126–592)^†^	392 (143–641)^†^	490 (192–788)^†^
Cortisol, ug/dL	11.0 (7.6–14.4)	16.7 (12.4–21.0)^†^	14.6 (9.7–19.6)	10.3 (5.6–15.0)	8.8 (7.0–10.7)
Total testosterone, ng/dL	514 (385–644)	604 (460–748)^†^	460 (332–587)^†^	535 (408–663)	544 (474–614)
Free testosterone, ng/dL	18.3 (13.6–22.9)	21.5 (16.3–26.7)^†^	16.3 (11.8–20.9)^†^	19.0 (14.4–23.7)	19.5 (17.0–22.0)
BDNF, pg/mL	254 (64–445)	565 (151–978)	437 (134–740)	332 (51–613)	97 (45–151)^†^
ALL-OUT					
Creatine kinase, U/L	259 (169–350)	346 (219–474)^†^	356 (236–476)^†^	332 (196–467)^†^	456 (181–731)^†^
Cortisol, ug/dL	13.9 (9.5–18.4)*	19.7 (14.9–24.5)^†^	19.5 (13.9–25.1)^†^	15.1 (11.4–18.8)	8.2 (6.7–9.7)^†^
Total testosterone, ng/dL	526 (491–562)	610 (539–682)^†^	493 (465–522)	504 (480–528)	570 (525–614)^†^
Free testosterone, ng/dL	18.3 (16.7–20.0)	20.7 (18.8–22.7)^†^	17.3 (15.7–18.8)	17.1 (16.0–18.2)	19.9 (18.7–20.9)
BDNF, pg/mL	298 (36–559)	632 (410–855)^†^	478 (173–783)	180 (41–319)	97 (55–139)^†^

BDNF, Brain-Derived Neurotrophic Factor; *Significantly different from control session (*p* ≤ 0.05); ^†^Significantly different from pre (*p* ≤ 0.05)

**Fig. 3:**
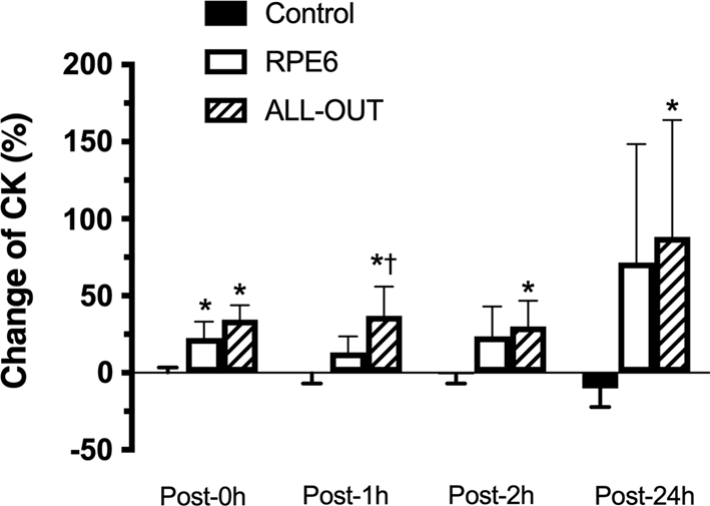
Percentage change in creatine kinase (CK) concentration post-FFT sessions with self-regulation of intensity (RPE6) and ALL-OUT and control session [mean (95% CI)]. *Significantly different from control (*p* ≤ 0.05); †Significantly different from RPE6 (*p* ≤ 0.05)

Figure 3 shows the percentage of change in creatine kinase concentration for each FFT session and the control session. The percentage of change post-1 h was significantly higher for all-out session compared to RPE6 session (*p* = 0.047). No other differences were observed in creatine kinase percentage change between all-out and RPE6 sessions. Effect size (ES) of the changes post-0 h are 0.00 for control, 0.65 for all-out and 0.28 for RPE6; ES Post-1 h are 0.02 for control, 0.69 for all-out and 0.14 for RPE6; ES Post-2 h are 0.01 for control, 0.54 for all-out and 0.24 for RPE6; ES Post-24 h are 0.13 for control, 0.85 for all-out and 0.51 for RPE6.

**Fig. 4:**
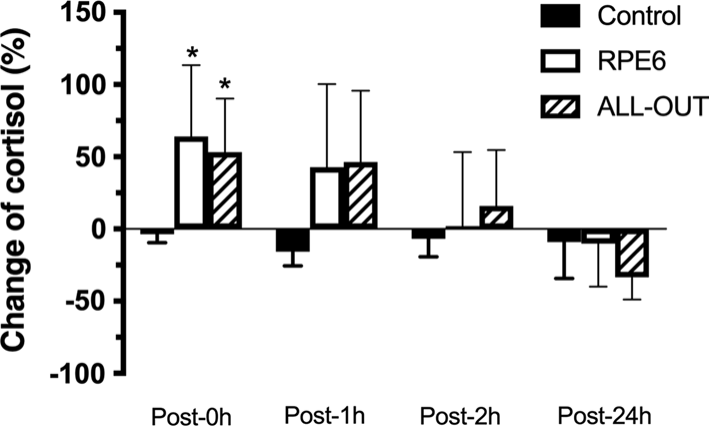
Percentage change in cortisol concentrations post-FFT sessions with self-regulation of intensity (RPE6) and ALL-OUT and control session [mean (95% CI)]. *Significantly different from control (*p* ≤ 0.05)

**Fig. 5:**
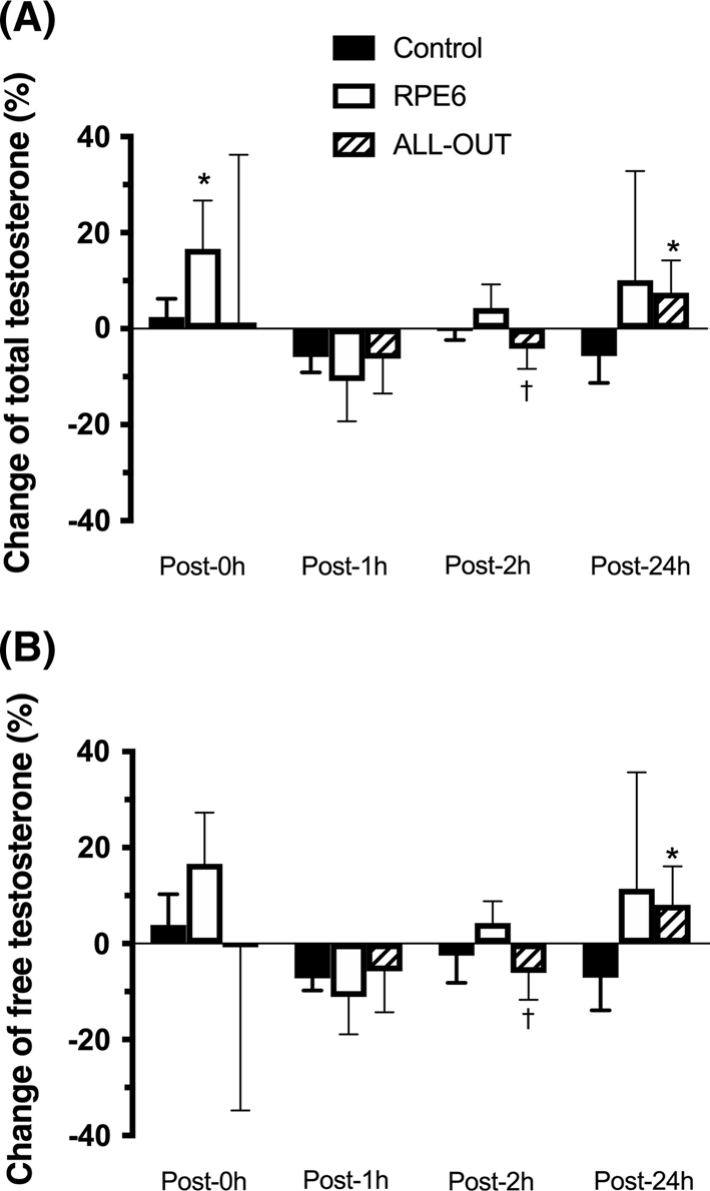
Percentage of change of total testosterone (**A**) and free testosterone (**B**) post-functional fitness sessions with self-regulation of intensity (RPE6) and ALL-OUT and control session [mean (95% CI)]. *Significantly different from control (*p* ≤ 0.05); †Significantly different from RPE6 (*p* ≤ 0.05)

**Fig. 6:**
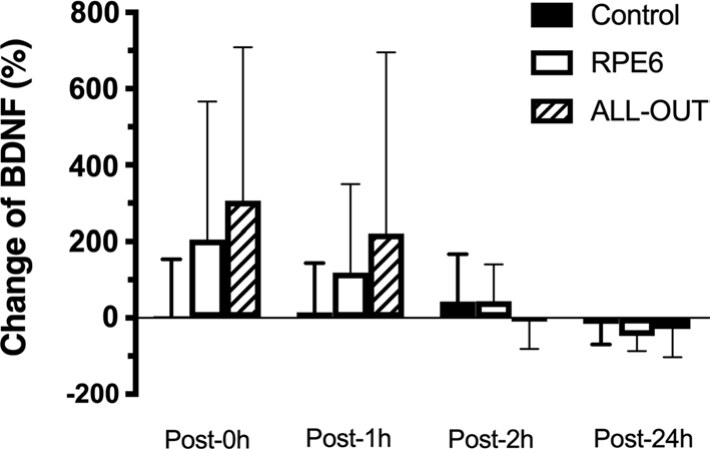
Percentage of change of Brain-Derived Neurotrophic Factor (BDNF) post-functional fitness sessions with self-regulation of intensity (RPE6) and ALL-OUT and control session [mean (95% CI)]

For cortisol concentrations, RPE6 and all-out sessions presented a significantly higher percentage change post-0 h compared to control session (Fig. 4). Effect size (ES) of the changes post-0 h are 0.13 for control, 1.07 for all-out and 1.34 for RPE6; ES Post-1 h are 0.68 for control, 0.81 for all-out and 0.73 for RPE6; ES Post-2 h are 0.35 for control, 0.21 for all-out and 0.18 for RPE6; ES Post-24 h are 0.65 for control, 1.31 for all-out and 0.67 for RPE6. The percentage change for total testosterone and free testosterone was significantly different (*p* ≤ 0.005 for total testosterone and *p* = 0.003 for free testosterone) only post-2 h between RPE-6 and all-out (Fig. 5). The Effect size (ES) of the changes for testosterone post-0 h was 0.17 for control, 1.26 for all-out and 0.57 for RPE6; ES Post-1 h are 0.40 for control, 1.02 for all-out and 0.37 for RPE6; ES Post-2 h are 0.05 for control, 0.71 for all-out and 0.14 for RPE6; ES Post-24 h are 0.40 for control, 0.88 for all-out and 0.21 for RPE6. No statistically significant differences (*p* > 0.05) were observed in the percentage change after the sessions for BDNF (Fig. 6). Effect size (ES) of the changes post-0 h are 0.20 for control, 1.33 for all-out and 0.59 for RPE6; ES Post-1 h are 0.07 for control, 0.67 for all-out and 0.56 for RPE6; ES Post-2 h are 0.42 for control, 0.40 for all-out and 0.05 for RPE6; ES Post-24 h are 0.27 for control, 1.21 for all-out and 1.33 for RPE6.

There were no significant correlations observed between blood lactate concentration or RPE at the end of the functional fitness sessions and creatine kinase, free testosterone, or total testosterone in the 24 h after functional fitness sessions. However, cortisol and BDNF concentrations after the FFT sessions were significantly correlated with blood lactate concentration, and RPE (Table 2).

**Table 2:** Correlations between cortisol and BDNF concentrations after the functional fitness sessions and blood lactate concentration, and ratings of perceived exertion after the functional fitness sessions

	Post-0 h	Post-1 h	Post-2 h	Post-24 h
Cortisol				
Blood lactate concentration	r = 0.58; *p* ≤ 0.01*	r = 0.53; *p* ≤ 0.01*	r = 0.35; *p* = 0.09	r = 0.15; *p* = 0.48
Ratings of perceived exertion	r = 0.75; *p* ≤ 0.01*	r = 0.71; *p* ≤ 0.01*	r = 0.54; *p* ≤ 0.01*	r = 0.21; *p* = 0.33
BDNF				
Blood lactate concentration	r = 0.51; *p* = 0.01*	r = 0.47; *p* = 0.02*	r = − 0.18; *p* = 0.40	r = − 0.57; *p* ≤ 0.01*
Ratings of perceived exertion	r = 0.47; *p* = 0.02*	r = 0.34; *p* = 0.10	r = − 0.32; *p* = 0.13	r = − 0.55; *p* ≤ 0.01*

BDNF, Brain-Derived Neurotrophic Factor; *statistically significant correlation

## Discussion

The major new finding from this study was that BDNF concentrations were greater post-exercise for the all-out session only. Similarly, the percentage change in creatine kinase at different time points after the sessions was only consistently higher than the control condition in the all-out session. These results suggest that intensity is a key factor in determining the responses elicited by FFT sessions. As the metabolic conditioning sessions of FFT are usually performed as “all-out” efforts, these results demonstrate that these types of sessions elicit a high level of hormonal and metabolic stress, and that manipulating the intensity of the metabolic conditioning sessions through the use of perceived exertion can lead to lower levels of hormonal and metabolic stress, potentially preventing negative outcomes associated with too much intense training, such as non-functional overreaching or overtraining.

Exercise-induced muscle damage (EIMD) can be attributed to the performance of unaccustomed exercise, or when the intensity, volume, and duration of the training stimulus is excessive to the participant, especially in poorly trained individuals [25]. Amongst other things, EIMD increases cell membrane permeability to muscle enzymes [26], causing leakage which is reflected in increased levels of several metabolic molecules in the interstitial fluid and blood [27, 28]. Furthermore, it is well known that all-out effort muscular work can potentially lead to muscle fiber impairment, that is correlated to increased serum creatine kinase concentration, with previous FFT studies demonstrating elevated creatine kinase levels post all-out sessions. Timón et al. [29] analyzed creatine kinase concentrations in two different training sessions, with the workout 1 consisting of as many rounds as possible of burpees and toes to bar, with the number of repetitions increasing for five minutes. Workout 2 consisted of three rounds of 20 repetitions of wall ball (9 kg) and 20 repetitions of power cleans in the shortest possible time. The creatine kinase post-24 h after training was approximately 673 and 864 U/L for workouts 1 and 2, respectively. Gomes et al. [30] also evaluated creatine kinase concentrations following a single workout (‘Cindy’—as many rounds as possible of 5 pull-ups, 10 push-ups, and 15 air squats in 20-min), and showed that creatine kinase concentrations increased post exercise (174.9 to 226.7 U/L) and remained elevated post-24 h (~ 270 U/L). The increase in the concentration occurred even though no external load was utilized, highlighting that the overall intensity of the session might be the key factor for creatine kinase changes.

In fact, when the intensity was likely reduced, the creatine kinase response to exercise was affected. Tibana et al. [11] analyzed the time-course response of creatine kinase following a FFT competition, where athletes were part of a team of three competitors. The results showed no statistical difference in creatine kinase concentration from baseline to post-competition. The fact that during team competitions in FFT the athletes perform a lower volume of repetitions when compared to individual competitions is a potential explanation for these results. In agreement with Tibana et al. [11], our results found a significant increase on creatine kinase percentage change post-24 h only after the all-out protocol. It is possible then, that a FFT session performed at a lower intensity (RPE6) can provide an adequate training stimulus, with a lower stress level to the participants. This would allow practitioners to manage the athletes’ training load throughout a training period, ensuring proper recovery during the week of training, potentially minimizing the negative effects associated with frequent all-out bouts of metabolic conditioning [6].

In general, the hormonal acute response is dependent upon the exercise intensity and is the most critical element to tissue remodeling [31]. In the male population, testosterone is a potent anabolic hormone that mediates protein accretion and enhances neural function [32]. A striking finding of the current study was that the total and free testosterone concentrations were lower post-2 h for all-out when compared to RPE6 session, suggesting a reduction in the secretory capacity linked to the gonadotropin action. Considering that the all-out session is characterized by greater energy demand and a higher level of neuromuscular fatigue, with the musculature often taken to the point of muscle failure, it is possible that this type of session needs a longer recovery before homeostasis can be restored and hormonal levels can be adjusted. Overall, the all-out session appeared to disrupt hormonal balance immediately after the session, and 2 h was not sufficient to restore testosterone levels. Nevertheless, the increased testosterone levels post-24 h compared to immediately after the sessions (post-0 h), may reflect a compensatory mechanism in response to the testosterone alteration in previous time-points.

Cortisol levels increased immediately post-session in both the RPE6 and all-out conditions, with no difference between sessions. This increase is likely related to enhanced glycogenolysis, gluconeogenesis, and protein catabolism to mobilize fuels for recovery and regeneration after exercise [33]. When investigating the time-course response of physiological, psychological and performance markers following a FFT competition, Tibana et al. [11] showed a significant decrease in cortisol concentrations after 48 h when compared to their pre-competition levels, indicating that cortisol release might have a later onset. Additionally, the authors did not observe any correlations between hormonal concentrations, metabolic responses, and immune variables with performance changes (countermovement jump), reinforcing the idea that cortisol changes in the short term are limited.

Prior studies showed that different exercise protocols can act as a stimulus to the hypothalamic-pituitary-adrenocortical axis, which in turn lead to increases in circulating cortisol levels [4, 34]. However, evidence suggests that cortisol levels increase at a rate relatively proportional to the exercise intensity yet reach a final level dependent upon the athlete’s training status, total duration of the exercise session, and the hormonal half-life [35–37]. Previous findings support this assertion. Jacks et al. [34] demonstrated that exercise sessions that lasted less than 40 min in duration elicited no significant differences in cortisol concentrations regardless of their intensity. Likewise, well trained individuals can tolerate a higher intensity of exercise prior to seeing an increase in cortisol concentrations, [36], which might partially explain our findings. Moreover, the release of cortisol typically is upstream of the immune system response [37]. Cortisol is also known to have potent anti-inflammatory effects, considering its role in maintaining neutrophilia, lymphopenia and cytokines bioavailability, which suggests that this hormone has a variety of effects on different functions [37].

Previous investigations that explored the BDNF kinetics after diverse exercise protocols have demonstrated acute transient increases in circulating levels during high-intensity when compared with low-intensity exercise [38, 39], with the highest concentrations occurring immediately post exercise. To the best of our knowledge, the present study is the first to analyze the role of the intensity on time-course changes in peripheral BDNF levels following FFT sessions. The current findings indicate that BDNF was greater (*p* = 0.002; ES = 1.33) post-0 h only for all-out session when compared to baseline concentrations, suggesting that intensity can modulate the BDNF amplitude response caused by FFT. Corroborating this finding, we found a positive correlation between blood lactate concentration during the sessions and BDNF values (post-0 h: r = 0.51; p = 0.01^*^; post-1 h: r = 0.47; *p* = 0.02^*^). Previous studies proposed that tropomyosin receptor kinase B receptor [40], Ca2^+^-stimulated intracellular signaling [41] and lactate concentrations [42] can modulate BDNF mRNA levels, which favors positive effects on neuroplasticity.

In a recent review, Müller et al. [43] highlighted a potential regulatory mechanism for the relationship between lactate and BDNF levels in response to exercise. It is well established that lactate promotes plasticity by potentiating NMDA glutamate receptor activity in neurons. Moreover, lactate upregulated intracellular NADH and calcium levels, which consequently can induce BDNF activation. This potential molecular basis to explain the contribution of the BDNF signaling pathway induced by lactate from astrocytes [43]. Moreover, lactate modulates PGC1α/FNDC5/BDNF pathway in response to exercise through SIRT1 activation [44]. However, while cortisol released during intense exercise might inhibit BDNF synthesis [44], the present study did not find a relationship between BDNF and cortisol responses. Further investigations are required to explain this result. The increases in BDNF concentration despite the changes in cortisol might algo suggest that other adjacent molecular pathways were involved in the changes in BDNF concentrations.

Recently, Ben-Zeev et al. [45] showed that a 3-month FFT program in middle-school adolescents was able to enhance short-term spatial learning, visual pattern separation, and inhibitory control. When comparing a FFT program to a walking intervention and a control group, Wilke et al. [46] suggested that FFT was more effective in improving working memory when compared to low intensity cardiovascular exercise. Thus, it is possible that the increase in BDNF levels after the all-out session reported in the present study can be one of the elements that links exercise to cognitive benefits [46].

Despite the interesting results of this study, some limitations need to be mentioned. First, the findings are limited to a relatively small (n = 8) sample of convenience, our specific athlete characteristics, and time frame. Moreover, the Tibana test does not contain all gymnastics movement, weightlifting and powerlifting exercises that are usually performed during the metabolic conditioning of FFT session. Future studies should include the investigation of regulatory molecules (e.g. catecholamine, neurotransmitters and glucocorticoid receptors) and immune variables that participate directly in the increased hormonal and metabolic responses following exercise in order to clarify adjacent mechanisms. In addition, whether longer periods of FFT sessions performed at different intensities (RPE6 and ALL-OUT, for example) will continue to produce further hormonal and metabolic adaptations, and if these changes are associated with modifications in muscle properties, strength, and functional ability remains a provocative hypothesis for further investigation.

## Conclusion

Taken together, the results demonstrate that the intensity at which the metabolic conditioning of FFT sessions are performed has a significant effect on hormonal and metabolic concentrations following exercise. All-out efforts can increase the acute concentration of BDNF and creatine kinase, while leading to an acute reduction in testosterone levels that return to baseline levels, and above, 24 h post training. When sessions are performed at a lower intensity (RPE6), a rapid recovery of physiological stress markers is seen when compared to an all-out session. The results also allow coaches and practitioners to improve their training programs based on the changes in hormonal and metabolic responses due to alterations in the intensity of the sessions. Future studies should evaluate the chronic effects of different training intensities in the markers of overreaching and overtraining syndrome in previously trained subjects.

## Data Availability

The datasets used and/or analyzed during this study are not publicly available because the authors do not have permission from the participants to publicly share their individual data, but are available from the corresponding author on reasonable request declarations.

## Abbreviations

AMRAP: As many rounds as possible
CK: Creatine kinase
BDNF: Brain derived neurotrophic factor
EIMD: Exercise-induced muscle damage
FFT: Functional-Fitness
RPE6: Self-regulation training intensity
